# Post-COVID-19 polyautoimmunity – Fact or coincidence: A case report

**DOI:** 10.3389/fmed.2023.1013125

**Published:** 2023-03-16

**Authors:** Ali Ibrahim Shorbagi, Abdulmunhem Obaideen, Majd Jundi

**Affiliations:** ^1^Clinical Sciences Department, College of Medicine, University of Sharjah, Sharjah, United Arab Emirates; ^2^Medical Diagnostic Imaging Department, University Hospital Sharjah, Sharjah, United Arab Emirates; ^3^Pathology Department, University Hospital Sharjah, Sharjah, United Arab Emirates

**Keywords:** SARS-CoV-2, COVID-19, autoimmune pancreatitis, ulcerative colitis, autoimmune hepatitis, immune-mediated hepatitis, case report

## Abstract

COVID-19 exhibits diverse and systemic clinical symptoms, much like systemic autoimmune diseases, and there are notable similarities in the immune responses seen in both conditions. There are rare reports of ulcerative colitis and autoimmune hepatitis triggered by COVID-19 infection. Reported herein is a case of a previously healthy patient who was diagnosed with chronic colitis resembling ulcerative colitis, autoimmune pancreatitis, and suspected immune-mediated hepatitis (AIH-like hepatitis) 2 months after a COVID-19 infection. A 33-year-old COVID-19-vaccinated male, presented with abdominal pain, nausea, and vomiting for 2 days. He also had bloody diarrhea that persisted for 2 months after recovering from a COVID-19 infection. A diagnosis of acute pancreatitis was confirmed by markedly elevated serum amylase and lipase and a CT scan of the abdomen. Colonoscopy and histopathology findings also confirmed a diagnosis of chronic colitis resembling ulcerative colitis (Mayo Endoscopy Subscore 3). Marked improvement in bloody diarrhea was observed within 72 h of treatment with IV prednisolone. MRI of the abdomen performed due to an unresolved clinical picture of pancreatitis revealed a bulky pancreas showing delayed diffuse homogenous enhancement, findings possibly consistent with autoimmune pancreatitis. Investigation for elevated liver transaminases showed high titers of antinuclear antibodies and anti-smooth muscle (anti-actin) antibodies while viral hepatitis markers were negative. The patient had already been started on steroid therapy before the lab results were available, with rapid normalization of liver enzymes following treatment. A liver biopsy was not performed. The patient is currently on mesalazine 4 gr/day, and azathioprine 100 mg/day – oral steroids had been tapered and discontinued. Seven months after the initial diagnosis, the patient remains symptom-free. A high level of suspicion for autoimmune disorders is required when assessing patients with a history of COVID-19 infection, although diagnostic pathways remain the same, with generally good response and remission rates to conventional treatment.

## Introduction

Polyautoimmunity is defined as the presence of more than one autoimmune disease in a single patient (1). Ulcerative colitis (UC) is an autoimmune inflammatory bowel disease (IBD) that may exhibit extra-intestinal manifestations in up to 45% of afflicted patients (2). Although strongly associated with primary sclerosing cholangitis, a recent report from a 13-year Danish registry of more than 22,000 patients does not mention an association with autoimmune hepatitis (AIH) (3). Similarly, autoimmune pancreatitis (AIP) is known to occur in association with UC, mostly as a complication of azathioprine treatment (2). Reported herein is a case of a COVID-19-vaccinated patient who was diagnosed with UC-like colitis, AIP, and suspected immune-mediated hepatitis **2** months after a COVID-19 infection.

## Case summary

**Figure 1:**
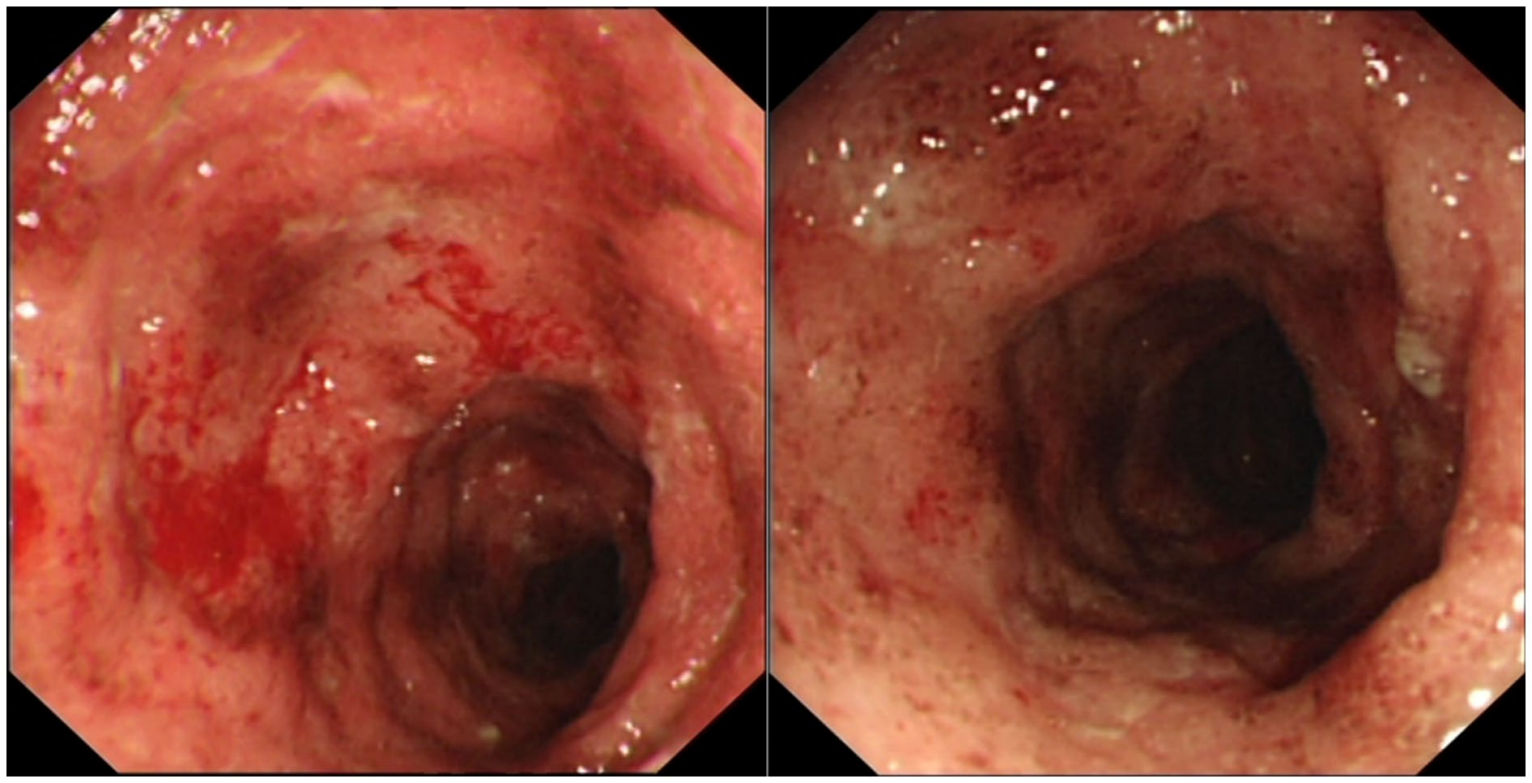
Colonoscopic images showing erythema and granularity of the examined colon mucosa with superficial exudated ulcerations, loss of normal vascular pattern and areas of spontaneous bleeding (Mayo Endoscopy Subscore 3).

**Figure 2:**
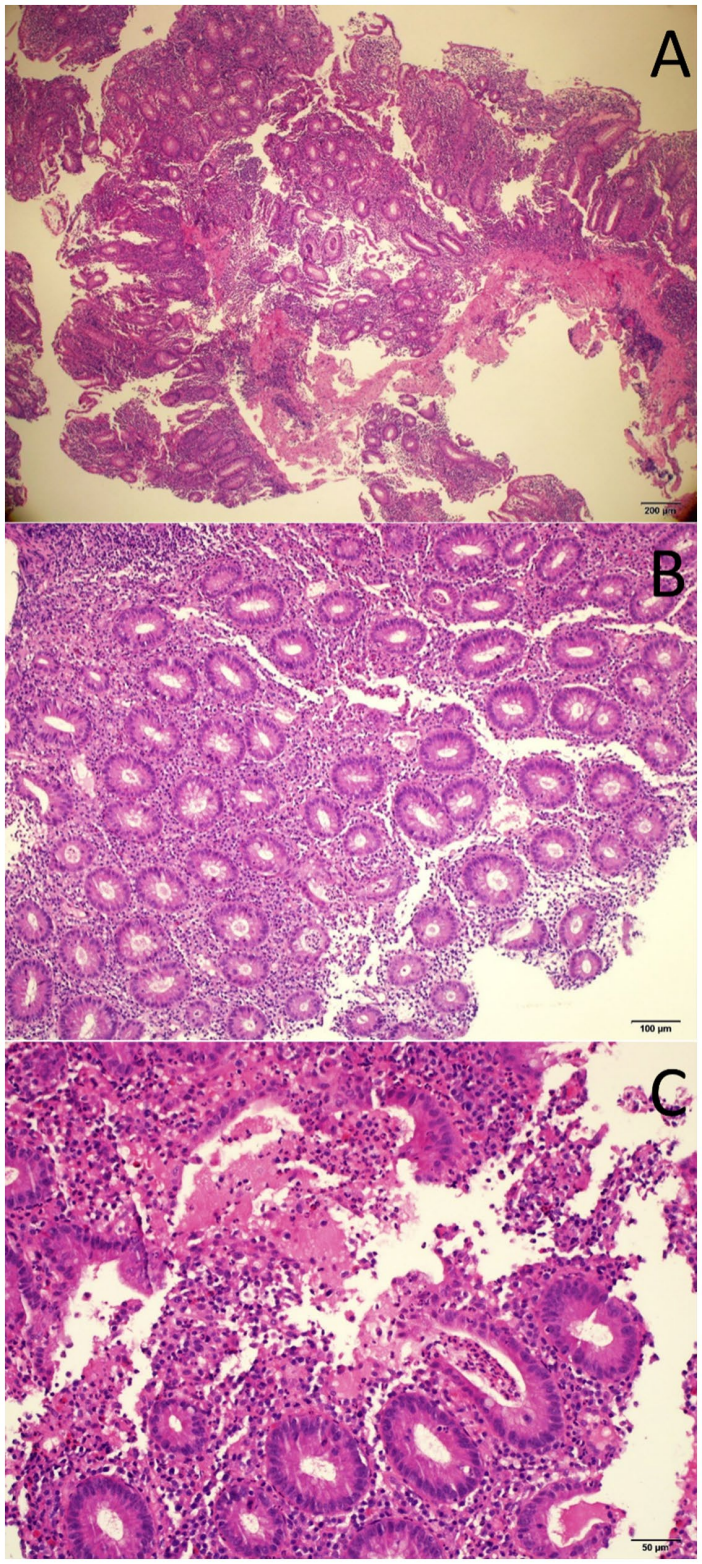
Histopathology images of endoscopic colon biopsy showing colonic mucosa with an inflammatory expansion of the lamina propria (**A**, H&E 40x), acute cryptitis (**B**, H&E 100X), and crypt abscesses (**C**, H&E 200X).

A 33-year-old-male, with no known comorbidities, presented to the emergency department on March 2022 with a two-day history of upper abdominal pain radiating to the back, nausea, and vomiting. He also complained of persistent diarrhea for the previous 2 months, with bloody stools for the previous two to 3 weeks. His diarrhea began soon after recovering from a mild COVID-19 infection in January 2022. He had 2 doses of Sinopharm^®^ vaccine in late 2020 and a single dose of the Pfizer^®^ vaccine in November 2021. He had received a short course of metronidazole 500 mg t.i.d 2 weeks prior with no obvious benefit. On examination, the patient appeared distressed, with a temperature of 37.1°C, pulse rate of 102 beats per minute, blood pressure of 117/77 mmHg, respiratory rate of 18 breaths per minute, and oxygen saturation of 96% on room air. His body mass index was 26.5 kg/m^2^. Initial blood results (summarized in Table 1) were significant for markedly elevated serum amylase, lipase, and CRP, with moderate elevation in ALT and AST (GGT and ALP were normal). Serum lipid profile and electrolytes were normal. CT scan of the abdomen without contrast showed peripancreatic fat stranding suggestive of early acute pancreatitis, mesenteric hyperemia, and multiple millimetric mesenteric lymph nodes. The patient did not give a history of alcohol consumption. His SARS-CoV-2 PCR test from a nasopharyngeal swab was negative. He was promptly hospitalized with a diagnosis of acute pancreatitis and for further investigation of chronic diarrhea with suspicion of UC. Ranson’s score on admission was “0.” He was kept on nil by mouth order and started on aggressive intravenous fluids with analgesia. Fecal calprotectin was markedly elevated and the result of a gastrointestinal PCR panel test was negative for *Clostridium difficile* and other pathogens. Colonoscopy showed erythema and granularity of the examined colon mucosa with superficial exudated ulcerations. There was a loss of haustrations and loss of normal vascular pattern with areas of spontaneous bleeding – Mayo Endoscopy Subscore 3 (Figure 1). On histopathology, colonic mucosa had relatively preserved architecture showing a marked expansion of the lamina propria by lymphoplasmacytic infiltrate (basal plasmocytosis) and mucosal neutrophilic infiltration with cryptitis and crypt abscesses (Figure 2). No granulomata were seen, and the findings were deemed consistent with UC-like colitis. The patient was started on 60 mg prednisolone IV and marked improvement in bloody diarrhea was observed within 72 h of treatment.

**Table 1:** Laboratory results.

	Result	Reference range
*Complete blood count*		
Hemoglobin	13.8 gr/dL	13.0–17.0 gr/dL
WBC count	13,600 /mm^3^	4,000–10,000/mm^3^
WBC differential	76.2% neutrophils, 14.8% lymphocytes, 0.9% eosinophils	
PLT count	365,000/mm^3^	150,000-410,000/mm^3^
*Biochemistry*		
Lipase	787 U/L	< 53 U/L
Amylase	461 U/L	< 34 U/L
CRP	121 mg/L	<10 mg/L
ALT	119 U/L	<49 U/L
AST	76 U/L	<48 U/L
GGT	45 U/L	<73 U/L
ALP	59 U/L	<116 U/L
Total cholesterol	2.2 mmol/L	< 5.2 mmol/L
Triglycerides	0.89 mmol/L	0.4–1.80 mmol/L
Calcium (ionized)	1.20 mmol/L	1.22–1.35 mmol/L
Total IgG	1,351 mg/dL	700–1,600 mg/dL
Total IgM	64 mg/dL	40–230 mg/dL
Serum IgG4	9 mg/dL	2–96 mg/dL
*Serology*		
HBs Antigen	Negative	
Anti-HAV IgM	Negative	
Anti-HCV	Negative	
ANA	1:1000 titer (nucleoplasm granular pattern)	
Anti-smooth muscle antibody (anti-actin)	65 U	0–19 U
Liver-kidney microsomal antibody	Negative	
Anti-mitochondrial M2 antibody:	Negative	
Soluble Liver Antigens (Anti-SLA) antibody	Negative	
Rheumatoid factor (RF)	Negative	
*Stool*		
Fecal calprotectin	> 800 μg/g	< 50 μg/g
GI PCR panel	Negative	
*Clostridium difficle* toxin	Negative	

**Figure 3:**
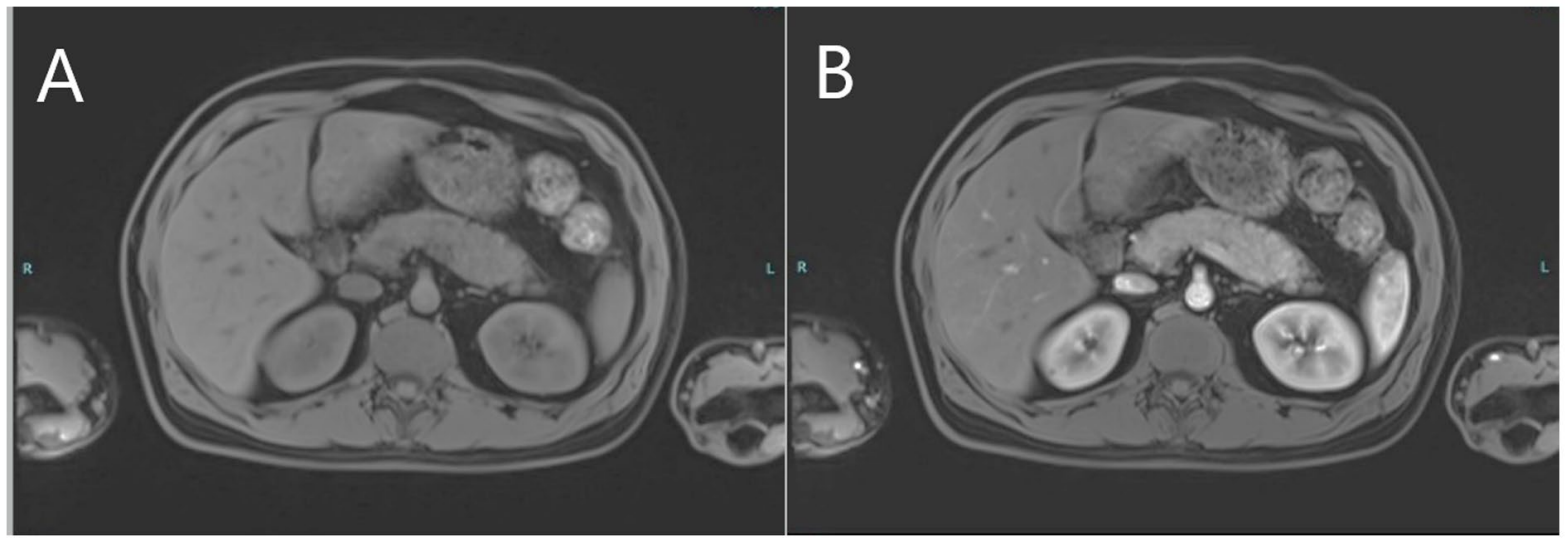
MRI images of the upper abdomen depicting pre-contrast bulky pancreas **(A)** and post-contrast delayed diffuse homogenous enhancement of the pancreas **(B)** consistent with autoimmune pancreatitis.

The patient continued to complain of recurring severe upper abdominal pain consistent with an unresolved clinical picture of pancreatitis as amylase and lipase levels remained high. Magnetic resonance cholangiopancreatography (MRCP) and contrast-enhanced MRI of the abdomen performed 10 days after initial presentation revealed a diffusely-enlarged, bulky pancreas showing delayed diffuse homogenous enhancement with no clear evidence of foci of necrosis or abscess formation (Figures 3A,B). There were no abnormalities suggestive of primary sclerosing cholangitis or pancreas divisum. Findings were deemed consistent with AIP. Serum IgG4 level was normal. In the presence of concomitant inflammatory bowel disease, the patient was diagnosed as “probable” type 2 AIP according to the International Consensus Diagnostic Criteria (ICDC) for AIP due to the absence of pancreatic biopsy (4).

**Figure 4:**
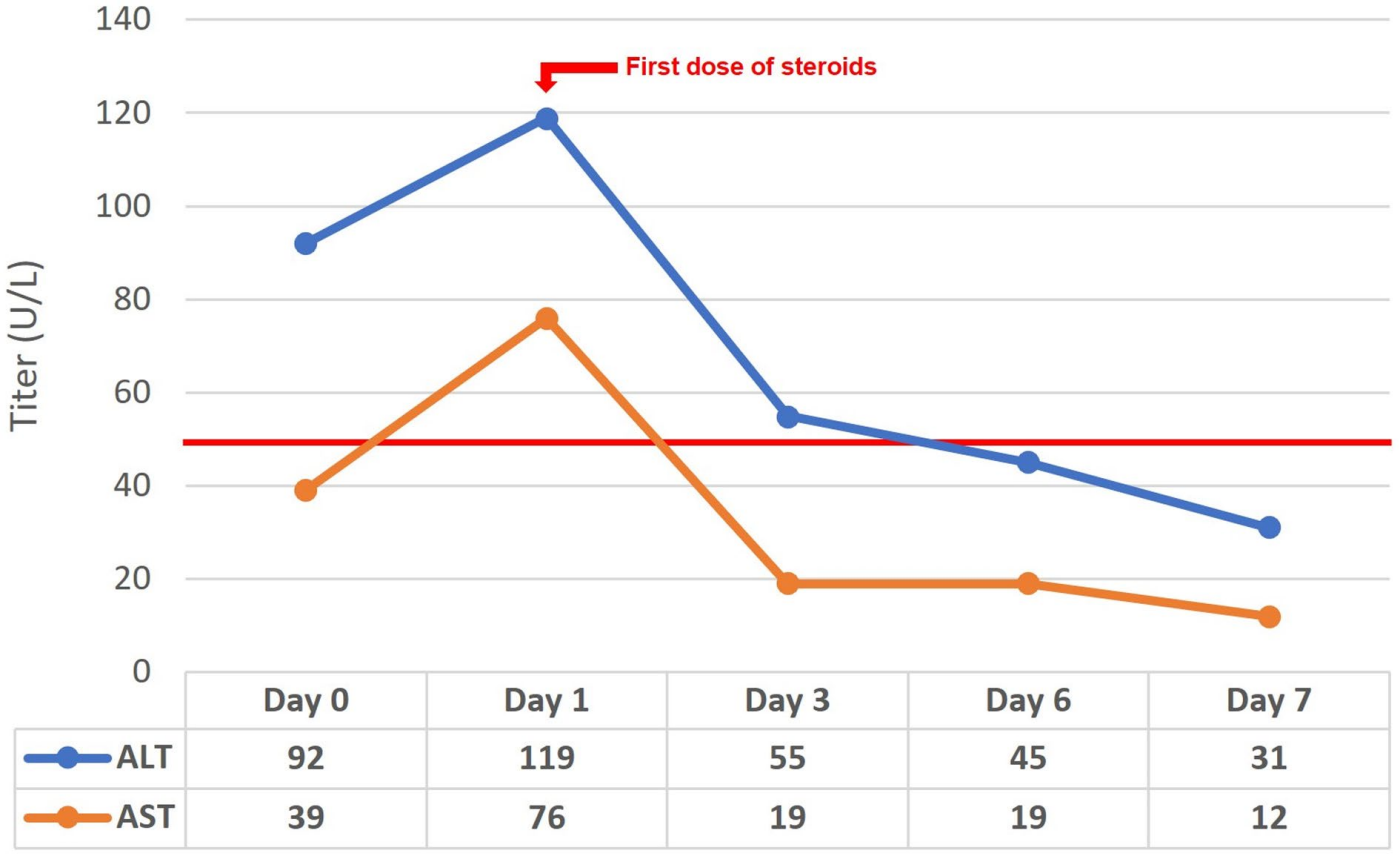
Change of liver enzymes over time.

For the investigation of elevated liver enzymes, viral hepatitis markers were negative. However delayed results of autoantibodies showed high titers of antinuclear antibodies and anti-smooth muscle (actin) antibodies. The liver-kidney microsomal and soluble liver antigen antibodies were negative. Serum IgG levels were also within normal limits. Unfortunately, the results of the liver autoimmune panel requested on admission were delayed, and the patient had already been started on steroid therapy for UC-like colitis and AIP. Liver enzyme follow-up before and after commencing steroids is depicted in Figure 4, with rapid normalization of enzymes following steroid treatment. The patient is currently on mesalazine 4 gr/day, and azathioprine 100 mg/day – oral steroids had been tapered, and eventually discontinued. Seven months after the initial diagnosis, the patient remains symptom-free having normal bowel movements 1–2 times per day, with no blood or mucus, and eating normally with no signs of pancreatitis.

## Discussion

The question that was raised with this case was whether the development of multiple autoimmune disorders could be related to the COVID-19 infection, in a previously healthy patient with no prior complaints.

The patient has UC-like colitis as confirmed by endoscopy and histopathology with typical features of mucosal neutrophilic infiltration, cryptitis, crypt abscesses, and basal plasmacytosis (5). A rapid response to steroid treatment also supports a diagnosis of immune-mediated (autoimmune) colitis. He also presented with a clinical picture of acute pancreatitis. He did not give a history of alcohol consumption or any medication that could potentially trigger acute pancreatitis, and there was no hypertriglyceridemia or hypercalcemia. Furthermore, MRCP and MRI confirmed the presence of a bulky pancreas showing delayed contrast enhancement, with no abnormalities such as pancreas divisum. Serum IgG4 was negative. In the presence of underlying UC-like colitis, it is reasonable to consider AIP as the most likely diagnosis.

The reported prevalence of AIP in IBD patients is 0.4–0.54%, while up to 40% of patients with confirmed type 2 AIP have an underlying IBD (6–8). The relationship between type 1 AIP and IBD is very weak, and it is widely believed that AIP that develops in patients with known IBD is most likely type 2. A definite diagnosis of type 2 AIP requires tissue confirmation of neutrophilic injury to the pancreatic ducts according to ICDC criteria. Unlike type 1 AIP, a “definite” diagnosis of type 2 AIP cannot be made without a biopsy even if they show typical imaging features of the disease; a diagnosis of type 2 AIP would be highly unlikely in the absence of an underlying IBD. According to a recent report, such patients who show a rapid response to steroids may be classified as having AIP “not-otherwise specified,” if there is no histopathological confirmation (8).

The presence of elevated liver transaminases with high titers of ANA and ASMA had raised a suspicion of AIH. Primary sclerosing cholangitis was not considered in this patient since GGT, ALP, and bilirubin levels were normal, and MRCP did not show any biliary tree abnormalities. Serum IgG levels were within normal limits. International guidelines recommend histological confirmation for AIH (9). However, the patient had already been on steroids for 10 days by the time the results of autoantibodies were obtained, and by then liver enzymes had already normalized. A liver biopsy was therefore not performed. Based on initial results, the patient does not fulfill the criteria for definite AIH-the patient’s score is consistent with “possible” AIH according to Simplified Criteria and Revised Original Score for the diagnosis of AIH (10, 11). Although it may be argued that drug-induced liver injury cannot be ruled out without a liver biopsy, especially without hypergammaglobulinemia, the patient had not received any recent medication that is strongly associated with liver injury. A recently proposed modification of the simplified score suggests that a diagnosis of AIH is “highly likely” if there is no history of drug use and there is a complete response to steroids, potentially alleviating the need for a liver biopsy (9). Normal IgG levels have been reported in up to 10% of patients with biopsy-confirmed AIH, and normal levels of IgG do not exclude a diagnosis of AIH (12). It may be argued that elevated transaminases in this patient could be coincidental, or even associated with acute pancreatitis. However, the high autoantibody titer, along with rapid normalization of liver enzymes following steroid treatment is strongly suggestive of immune-mediated liver injury (AIH-like hepatitis). The concurrence of ANA and SMA has a specificity of 99%, and diagnostic accuracy of 74% for AIH, and SMA positivity is almost always associated with an underlying condition, most notably type 1 AIH (13).

What makes this case interesting is the association of three concomitant autoimmune conditions in a previously healthy individual 2 months after a COVID-19 infection. Such an association has not been previously reported in the literature. COVID-19 is known to present in heterogeneous and systemic clinical manifestations. Several similarities have been reported in the immune responses in COVID-19 and autoimmune diseases (14). The organ damage in COVID-19 appears to be largely immune-mediated, similar to autoimmune diseases. It is believed that self-tolerance of host antigens through molecular mimicry results in the development of autoantibodies which contribute to the development of organ-specific or systemic autoimmune disorders. Recent studies have found a strong correlation between COVID-19 infection and the development of autoimmunity (15–17). Alterations induced by the virus, such as phosphorylation and ubiquitination of proteins and RNA, lead to significant molecular changes that can produce autoantigenic proteins, causing short-term and long-lasting autoimmune responses. These molecular changes appear to be closely linked to various COVID-19 symptoms and provide insight into the host’s response to the virus. Moreover, these alterations have been implicated in host cell death, autoimmune responses mediated by autoAg-DS complexes, and the eventual onset of autoimmune diseases.

There are rare reports of UC and AIH triggered by either COVID-19 infection, or by mRNA vaccines (18–21). In a recent report on UC triggered by a mild COVID-19 infection, authors cited gut microbiota modification and dysregulation of the normal flora bacteria due to SARS-CoV-2 infection may trigger inflammation due to angiotensin II overexpression (22). COVID-19-associated molecular mimicry may activate an immune response to antigenic epitopes distinct from the disease-causing epitopes, activating T cells by bystander activation, or exposing cryptic epitopes, which could be the cause of *de novo* UC or reactivation of preexisting disease. Although several cases of acute pancreatitis have been reported in association with COVID-19 infection, particularly in the acute phase, no report of type 2 AIP could be encountered in the literature (23).

## Conclusion

A high level of suspicion for autoimmune disorders is required when assessing patients with a history of COVID-19 infection, although as several reports indicated, diagnostic pathways remain the same, with generally good response and remission rates comparable to conventional treatment. Further research is required to further elucidate the mechanisms behind COVID-19-associated autoimmunity.

## Data availability statement

The original contributions presented in the study are included in the article/supplementary material, further inquiries can be directed to the corresponding author.

## Ethics statement

Ethical review and approval was not required for the study on human participants in accordance with the local legislation and institutional requirements. Written informed consent from the patients was not required to participate in this study in accordance with the national legislation and the institutional requirements. Written informed consent was obtained from the individual for the publication of any potentially identifiable images or data included in this article.

## Author contributions

All authors listed have made a substantial, direct, and intellectual contribution to the work and approved it for publication.

## Conflict of interest

The authors declare that the research was conducted in the absence of any commercial or financial relationships that could be construed as a potential conflict of interest.

## Publisher’s note

All claims expressed in this article are solely those of the authors and do not necessarily represent those of their affiliated organizations, or those of the publisher, the editors and the reviewers. Any product that may be evaluated in this article, or claim that may be made by its manufacturer, is not guaranteed or endorsed by the publisher.

## References

[1] Rojas-Villarraga A Amaya-Amaya J Rodriguez-Rodriguez A Mantilla RD Anaya JM. Introducing polyautoimmunity: secondary autoimmune diseases no longer exist. Autoimmune Dis. (2012) 2012:254319. doi: 10.1155/2012/254319, PMID: 22454759 PMC3290803

[2] Almeida P Almeida C Gompertz M Berger Z. Association between autoimmune pancreatitis and ulcerative colitis: a report of 12 patients. Rev Esp Enferm Dig. (2020) 112:682–7. doi: 10.17235/reed.2020.6677/2019, PMID: 32578999

[3] Vadstrup K Alulis S Borsi A Jørgensen TR Nielsen A Munkholm P. Extraintestinal manifestations and other comorbidities in ulcerative colitis and Crohn disease: a Danish Nationwide registry study 2003–2016. Crohn's Colitis 360. (2020) 2, 2:otaa070. doi: 10.1093/crocol/otaa070, PMID: 36776496 PMC9802257

[4] Shimosegawa T Chari ST Frulloni L Kamisawa T Kawa S Mino-Kenudson M. International Association of Pancreatology. International consensus diagnostic criteria for autoimmune pancreatitis: guidelines of the International Association of Pancreatology. Pancreas. (2011) 40:352–8. doi: 10.1097/MPA.0b013e3182142fd2 21412117

[5] Lang-Schwarz C Angeloni M Agaimy A Atreya R Becker C Dregelies T. Validation of the “inflammatory bowel disease-distribution, chronicity, activity [IBD-DCA] score” for ulcerative colitis and Crohn’s disease. J Crohns Colitis. (2021) 15:1621–30. doi: 10.1093/ecco-jcc/jjab055, PMID: 33773497 PMC8495487

[6] Ueki T Kawamoto K Otsuka Y Minoda R Maruo T Matsumura K. Prevalence and clinicopathological features of autoimmune pancreatitis in Japanese patients with inflammatory bowel disease. Pancreas. (2015) 44:434–40. doi: 10.1097/MPA.0000000000000261, PMID: 25469544

[7] Park SH Kim D Ye BD Yang SK Kim JH Yang DH. The characteristics of ulcerative colitis associated with autoimmune pancreatitis. J Clin Gastroenterol. (2013) 47:520–5. doi: 10.1097/MCG.0b013e31827fd4a2, PMID: 23426453

[8] Zen Y. Type 2 autoimmune pancreatitis: consensus and controversies. Gut Liver. (2022) 16:357–65. doi: 10.5009/gnl210241, PMID: 34670874 PMC9099380

[9] Shorbagi AI. Is it time to get rid of the biopsy mandate in adults with suspected autoimmune hepatitis? Lessons from the COVID-19 pandemic. Liver Int. (2022) 42:480–1. doi: 10.1111/liv.15137, PMID: 34919326

[10] Hennes EM Zeniya M Czaja AJ Parés A Dalekos GN Krawitt EL. International autoimmune hepatitis group. Simplified criteria for the diagnosis of autoimmune hepatitis. Hepatology. (2008) 48:169–76. doi: 10.1002/hep.22322, PMID: 18537184

[11] Alvarez F Berg PA Bianchi FB Bianchi L Burroughs AK Cancado EL. International autoimmune hepatitis group report: review of criteria for diagnosis of autoimmune hepatitis. J Hepatol. (1999) 31:929–38. doi: 10.1016/s0168-8278(99)80297-9, PMID: 10580593

[12] Hartl J Miquel R Zachou K Wong GW Asghar A Pape S. Features and outcome of AIH patients without elevation of IgG. JHEP Rep. (2020) 2:100094. doi: 10.1016/j.jhepr.2020.100094, PMID: 32280942 PMC7139106

[13] Czaja AJ. Performance parameters of the conventional serological markers for autoimmune hepatitis. Dig Dis Sci. (2011) 56:545–54. doi: 10.1007/s10620-010-1501-1, PMID: 21127976

[14] Liu Y Sawalha AH Lu Q. COVID-19 and autoimmune diseases. Curr Opin Rheumatol. (2021) 33:155–62. doi: 10.1097/BOR.0000000000000776, PMID: 33332890 PMC7880581

[15] Wang JY Zhang W Roehrl MW Roehrl VB Roehrl MH. An autoantigen profile of human A549 lung cells reveals viral and host etiologic molecular attributes of autoimmunity in COVID-19. J Autoimmun. (2021) 120:102644. doi: 10.1016/j.jaut.2021.102644, PMID: 33971585 PMC8075847

[16] Wang JY Roehrl MW Roehrl VB Roehrl MH. A master autoantigen-ome links alternative splicing, female predilection, and COVID-19 to autoimmune diseases. J Transl Autoimmun. (2022) 5:100147. doi: 10.1016/j.jtauto.2022.100147, PMID: 35237749 PMC8872718

[17] Wang JY Zhang W Roehrl VB Roehrl MW Roehrl MH. An autoantigen atlas from human lung HFL1 cells offers clues to neurological and diverse autoimmune manifestations of COVID-19. Front Immunol. (2022) 13:831849. doi: 10.3389/fimmu.2022.831849 35401574 PMC8987778

[18] Aydın MF Taşdemir H. Ulcerative colitis in a COVID-19 patient: a case report. Turk J Gastroenterol. (2021) 32:543–7. doi: 10.5152/tjg.2021.20851, PMID: 34405821 PMC8975413

[19] Imperatore N Bennato R D'Avino A Lombardi G Manguso F. SARS-CoV-2 as a trigger for De novo ulcerative colitis. Inflamm Bowel Dis. (2021) 27:e87–8. doi: 10.1093/ibd/izab040, PMID: 33616182 PMC7928823

[20] Kabaçam G Wahlin S Efe C. Autoimmune hepatitis triggered by COVID-19: a report of two cases. Liver Int. (2021) 41:2527–8. doi: 10.1111/liv.15044, PMID: 34478591 PMC8662284

[21] Hong JK Chopra S Kahn JA Kim B Khemichian S. Autoimmune hepatitis triggered by COVID-19. Intern Med J. (2021) 51:1182–3. doi: 10.1111/imj.15420, PMID: 34278694 PMC8447478

[22] Fonseca Mora MC Abushahin A Gupta R Winters H Khan GM. Severe ulcerative colitis as a complication of mild COVID-19 infection in a vaccinated patient. Cureus. (2022) 14:e25783. doi: 10.7759/cureus.25783, PMID: 35812630 PMC9270872

[23] Correia de Sá T Soares C Rocha M. Acute pancreatitis and COVID-19: a literature review. World. J Gastrointest Surg. (2021) 13:574–84. doi: 10.4240/wjgs.v13.i6.574, PMID: 34194615 PMC8223706

